# A survey of overall life satisfaction and its association with breast diseases in Chinese women

**DOI:** 10.1002/cam4.565

**Published:** 2015-12-07

**Authors:** Aili Bai, Haixin Li, Yubei Huang, Xueou Liu, Ying Gao, Peishan Wang, Hongji Dai, Fengju Song, Xishan Hao, Kexin Chen

**Affiliations:** ^1^ Departments of Epidemiology and BiostatisticsKey Laboratory of Cancer Prevention and Therapy, TianjinNational Clinical Research Center of CancerTianjin Medical University Cancer Institute and HospitalTianjin300060China

**Keywords:** Benign breast diseases, breast cancer, detection rate, lifestyle, overall life satisfaction, physical examination

## Abstract

To investigate the association between overall life satisfaction and healthy lifestyle, knowledge of breast cancer, physical examination, and detection rate of breast cancer and benign breast disease in Chinese women. In a multicentered breast disease screening program in China, we enrolled 33,057 women aged 45–65 years without prior diagnosis of breast cancer. After completing an epidemiological questionnaire, all participants were examined by clinical breast examination, breast ultrasound, and mammography independently. All breast cancer cases and a selected sample of benign breast diseases were confirmed pathologically. Univariate and multivariate odds ratios (ORs) and 95% confidence intervals (CIs) were calculated to estimate the association between life satisfaction and lifestyle, knowledge of breast cancer, physical examination, and detection rate of breast diseases. Overall life satisfaction was positively associated with women's healthy lifestyle. Compared with less satisfied women, satisfied women were less likely to be smokers (OR = 0.54; 95% CI: 0.47–0.62), have more exercise (OR = 1.49; 95% CI: 1.26–1.75), eat less fried (OR = 0.60; 95% CI: 0.50–0.71), smoked (OR = 0.54, 95% CI: 0.47–0.63), pickled (OR = 0.66, 95% CI: 0.55–0.79), and grilled (OR = 0.63, 95% CI: 0.54–0.74) foods. Satisfied women were more likely to have knowledge of breast cancer (OR = 1.48, 95% CI: 1.29–1.70), and have regular physical examinations (OR = 1.11, 95% CI: 1.01–1.12). Compared to less satisfied women, we found significantly lower detection rate of benign breast diseases (OR = 0.90, 95% CI: 0.82–0.99), and lower but nonsignificant detection rate of breast cancer (OR = 0.66, 95% CI: 0.35–1.25) in satisfied women. Women with a higher overall life satisfaction are more likely to have healthy lifestyle, knowledge of breast cancer, and regular physical examination, thus resulting in a lower detection rate of breast diseases in screening.

## Introduction

Breast cancer has become the most frequently diagnosed malignant tumor in women worldwide, and more than 1.6 million Chinese women would be diagnosed with breast cancer each year 1. Benign breast diseases (BBDs) are much more common and have a close relationship with breast cancer. Certain subtypes of BBD, particularly atypical hyperplasia, are highly transferable to breast cancer 2, 3.

The association between psychological factors and risk of breast cancer has been established 4, but the mechanism of the association is not clear. It is proposed that the psychological factors can directly interfere with the functions of neuroendocrine and immune system, thus influencing the risk of breast cancer 5. For example, stress‐related factors may decrease the activity of natural killer cell, which is an important part of the immune system for tumor surveillance 6. It is notable that the changes in lifestyle (smoking, alcohol consumption, diet, exercise) and health cognition (seeking for health care) are also regarded as an indirect mediating link between psychological factors and breast cancer development 5. However, to the best of our knowledge, there are no studies addressing the associations between psychological factors and lifestyle, knowledge of breast cancer, and physical examination. Only a few case–control studies investigated BBD in relation to psychological factors, while probably because of insufficient sample size, most studies failed to find the roles of adverse life experience/stress 7, 8, anxiety or depression 9, 10 in increasing the risk of BBD.

Our study was designed to evaluate the association between overall life satisfaction and lifestyle, knowledge of breast cancer as well as physical examination, and to estimate the relationship between overall life satisfaction and the detection rates of breast cancer and benign breast diseases among Chinese females based on the Multimodality Independent Screening Trial (MIST) in China. Overall life satisfaction in this study was regarded as a comprehensive concept used to evaluate subjective well‐being of life 11. We hypothesized that life satisfaction may positively impact on breast cancer by promoting healthy lifestyle and/or knowledge of breast cancer and regular physical examinations.

## Methods

### Study population

Our study is based on data from the MIST, a multicentered breast disease screening program performed by the Chinese Anti‐Cancer Association (CACA) from 2008 to 2010. The screening procedure has been presented in detail elsewhere 12. In brief, multistage clustered sampling methods were used for selection of women from 45 years old to 65 years old without malignant breast tumor history in five cities (from larger to smaller, Beijing, Tianjin, Shenyang, Nanchang, and Feicheng) in China. 3000–5000 women were screened in each city and about 35,000 asymptomatic women entered the screening. Before screening, a face‐to‐face interviewed questionnaire was used to collect information on overall life satisfaction, demographic factors, reproductive factors, family history of cancer, lifestyle factors, knowledge of breast cancer, physical examinations, and personality factors. Each participant was examined by clinical breast examination (CBE), breast ultrasound (BUS), and mammography (MAM) independently. Participants were suggested to do a needle aspiration biopsy or surgery if their primary physicians considered suspicious or highly suggestive of a malignancy according to the three methods. Participants would be considered as patients with benign breast disease (BBD) if diagnosed by at least one examination. All breast cancer patients and a selected sample of BBD cases (*n* = 181) were confirmed by pathological examination. A total of 33,057 women are included in the final analysis. This study was approved by the Tianjin Medical University Cancer Institute and Hospital (TMUCIH) Institutional Review Board. All the participants had written informed consent before breast disease screening.

### Main variables

Overall life satisfaction was collected from a question “How would you rate your satisfaction with overall life”. A single response was selected from the five categories: very satisfied, satisfied, just so‐so, less satisfied, and not satisfied. In the analysis, we combine the first two categories as “satisfied”, and the last three categories as “less satisfied”.

Lifestyle factors of women in this study mainly include smoking (yes, no), alcohol consumption (yes, no), physical activity (times/week), and diet. Diet measures consist of diet patterns (proportion of vegetables and meat) and frequency of unhealthy foods (fried, smoked, pickled, and grilled) intake weekly. Information on diet and physical activity are only available in the 2009 and 2010 questionnaire.

Knowledge of breast cancer was measured by the question: do you have knowledge of breast cancer, such as risk factors or prevention (yes, a little, and no)? Sources of your breast cancer knowledge included newspaper and books, radio and television, health education, friends or relatives with breast cancer, or other ways. The number of sources is used for analysis. Physical examination was measured by the question “do you participate in regular physical examinations (yes, no)”.

In this study, demographic factors include region, age, ethnicities, education level, occupation, height, body weight, household income, source of health insurance, marital status, age at marriage, miscarriages, number of children, family history of cancer, and self‐assessment of personality, health condition, interpersonal relationship, and major setbacks or unfortunate events. Height and weight are measured by investigators, and body mass index (BMI) is calculated using the following formula: BMI = (weight in kg)/(height in m^2^). Family history of cancer is defined as cancer occurring in first‐degree relatives, including grandparents, parents, sisters and brothers, and children.

### Statistical analysis

Firstly, unconditional univariate and multivariate logistic regression are used to estimate the association between overall life satisfaction and potential‐related factors including region (Beijing, Tianjin, Shenyang, Nanchang, and Feicheng), age (<55 years, ≥55 years), ethnicities (Han, minority), education level (primary or below, middle school, and college or above), occupation (yes, no), household income (low, middle, and high), marital status (married, others including unmarried, divorced, widowed, and separated), age at marriage (≤20 years, 21–30 years, and >30 years), health insurance (self‐paying, medical insurance, and free medical care), BMI (<18.5, 18.5–23.9, 24.0–27.9, and ≥28.0), characters (extrovert, introvert, and both), health condition (good, medium, and bad), interpersonal relationship (good, medium, and bad), major setbacks or unfortunate events (yes, no), number of children (0, 1, and >1), miscarriages (yes, no), and family history of cancer (yes, no).

Secondly, we estimate the association between overall life satisfaction and lifestyle, the association between overall life satisfaction and knowledge of breast cancer, physical examination, and the association between overall life satisfaction and detection rates of breast cancer and benign breast diseases by using univariate‐ and multivariate‐adjusted models, including factors significantly associated with overall life satisfaction in prior analysis.

Data analysis is performed with SPSS 17.0 (SPSS Inc., Chicago, IL). Odds ratios (ORs) and 95% confidence intervals (CIs) are provided in the results and all *P*‐values are two sided.

## Results

### Factors associated with overall life satisfaction

Among the 33,057 women included in the analysis, 30,149 (91.2%) are reported satisfied with overall life, while 2908 (8.8%) are reported less satisfied. Table 1 shows the factors associated with overall life satisfaction. In the multivariate analysis, region, age, occupation, household income, age at marriage, health insurance, personal character, health condition, interpersonal relationship, and major unfortunate events are significantly associated with overall life satisfaction. Women living in smaller cities tend to be more satisfied. Compared to women living in Beijing, women living in Tianjin (OR = 1.92; 95% CI: 1.62–2.28), Shenyang (OR = 2.00; 95% CI: 1.74–2.31), Nanchang (OR = 2.49; 95% CI: 2.16–2.86), and Feicheng (OR = 7.98; 95% CI: 5.87–10.85) are more satisfied with life. Older women (≥55 years) are more satisfied than younger women (<55 years) (OR = 1.32; 95% CI: 1.17–1.49). Additionally, women who have occupation (OR = 1.28; 95% CI: 1.14–1.43), high household income (OR = 2.11; 95% CI: 1.79–2.48), and free health care (OR = 1.33; 95% CI: 1.10–1.62) are more satisfied than women without occupation, low income, and self‐paying health care, respectively. Women with extrovert character (OR = 2.13; 95% CI: 1.88–2.43), good health condition (OR = 3.93; 95% CI: 3.11–4.95), and good interpersonal relationship (OR = 11.24; 95% CI: 6.34–19.92) are more satisfied compared to women with introvert character, bad health condition, and bad interpersonal relationship, respectively. An older age at marriage (>30 years) is negatively associated with overall life satisfaction compared to regular age at marriage (21–30) (OR = 0.75; 95% CI: 0.60–0.94). Major unfortunate events (OR = 0.36; 95% CI: 0.32–0.40) are negatively associated with overall life satisfaction. However, ethnicities, education levels, marital status, number of children, BMI, miscarriages, and family history of cancer are not found to be associated with overall life satisfaction in this study.

**Table 1:** Factors affecting overall life satisfaction in Chinese women

Factors (*N*)	Less satisfied (%) (2908)	Satisfied (%) (30,149)	Univariate		Multivariate^a^	
			OR (95% CI)	*P*	OR (95% CI)	*P*
Region (33,057)						
Beijing	786 (27.0)	4633 (15.4)	1.00		1.00	
Tianjin	623 (21.4)	7161 (23.8)	1.95 (1.74, 2.18)	<0.001	1.92 (1.62, 2.28)	<0.001
Shenyang	651 (22.4)	7321 (24.3)	1.91 (1.71, 2.13)	<0.001	2.00 (1.74, 2.31)	<0.001
Nanchang	796 (27.4)	8059 (26.7)	1.72 (1.55, 1.91)	<0.001	2.49 (2.16, 2.86)	<0.001
Feicheng	52 (1.8)	2975 (9.9)	9.71 (7.30, 12.90)	<0.001	7.98 (5.87, 10.85)	<0.001
Age (33,057)						
<55	2319 (79.8)	22,358 (74.2)	1.00		1.00	
≥55	589 (20.2)	7791 (25.8)	1.37 (1.25, 1.51)	<0.001	1.32 (1.17, 1.49)	<0.001
Ethnicities (28,866)						
Han	2541 (98.2)	25,717 (97.9)	1.00		1.00	
Minority	47 (1.8)	561 (2.1)	1.18 (0.87, 1.59)	0.282	1.36 (0.97, 1.92)	0.076
Education level (32,950)						
Middle school	1934 (66.9)	18,583 (61.8)	1.00		1.00	
Primary or below	161 (5.6)	1988 (6.6)	1.29 (1.09, 1.52)	0.003	1.10 (0.89, 1.36)	0.388
College or above	798 (27.6)	9486 (31.6)	1.24 (1.14, 1.35)	<0.001	1.04 (0.93, 1.16)	0.496
Occupation (32,928)						
No	1116 (38.6)	9626 (32.0)	1.00		1.00	
Yes	1774 (61.4)	20,412 (68.0)	1.33 (1.23, 1.44)	<0.001	1.28 (1.14, 1.43)	<0.001
Household income^b^ (32,546)						
Low	1118 (39.3)	8291 (27.9)	1.00		1.00	
Medium	1421 (49.9)	16,380 (55.2)	1.55 (1.43, 1.69)	<0.001	1.51 (1.36, 1.68)	<0.001
High	309 (10.8)	5027 (16.9)	2.19 (1.92, 2.50)	<0.001	2.11 (1.79, 2.48)	<0.001
Marital status (33,044)						
Single^c^	39 (1.3)	185 (0.6)	1.00		1.00	
Married	2866 (98.7)	29,954 (99.4)	2.20 (1.56, 3.12)	<0.001	1.32 (0.56, 3.14)	0.526
Age at marriage (32,436)						
21–30 years	2594 (91.3)	27,486 (92.9)	1.00		1.00	
≤20 years	103 (3.6)	1140 (3.9)	1.05 (0.85, 1.28)	0.678	1.11 (0.87, 1.41)	0.409
>30 years	145 (5.1)	968 (3.3)	0.63 (0.53, 0.75)	<0.001	0.75 (0.60, 0.94)	0.014
Health insurance (32,717)						
Self‐paying	488 (17.0)	4116 (13.8)	1.00		1.00	
Insurance	2106 (73.5)	22,375 (75.0)	1.26 (1.14, 1.40)	<0.001	1.05 (0.92, 1.19)	0.486
Free	270 (9.4)	3362 (11.3)	1.48 (1.26, 1.73)	<0.001	1.33 (1.10, 1.62)	0.003
BMI (32,733)						
18.5–23.9	1575 (54.7)	16,209 (54.3)	1.00		1.00	
<18.5	72 (2.5)	712 (2.4)	0.96 (0.75, 1.23)	0.752	0.96 (0.73, 1.27)	0.775
24.0–27.9	1011 (35.1)	10,516 (35.2)	1.01 (0.93, 1.10)	0.801	0.95 (0.86, 1.05)	0.277
≥28.0	221 (7.7)	2417 (8.1)	1.06 (0.92, 1.23)	0.418	1.01 (0.84, 1.21)	0.957
Personal character (33,016)						
Introvert	630 (21.7)	3675 (12.2)	1.00		1.00	
Middle	1246 (42.9)	11,628 (38.6)	1.60 (1.44, 1.77)	<0.001	1.43 (1.26, 1.62)	<0.001
Extrovert	1028 (35.4)	14,809 (49.2)	2.47 (2.22, 2.74)	<0.001	2.13 (1.88, 2.43)	<0.001
Health condition (32,994)						
Bad	177 (6.1)	462 (1.5)	1.00		1.00	
Medium	1429 (49.3)	9755 (32.4)	2.62 (2.18, 3.14)	<0.001	2.64 (2.10, 3.32)	<0.001
Good	1295 (44.6)	19,876 (66.0)	5.88 (4.90, 7.06)	<0.001	3.93 (3.11, 4.95)	<0.001
Interpersonal relationship (32,953)						
Bad	32 (1.1)	36 (0.1)	1.00		1.00	
Medium	1275 (44.0)	5755 (19.1)	4.01 (2.48, 6.48)	<0.001	4.18 (2.36, 7.47)	<0.001
Good	1589 (54.9)	24,266 (80.7)	13.57 (8.41, 21.91)	<0.001	11.24 (6.34, 19.92)	<0.001
Major setback/unfortunate events (32,800)						
Yes	712 (24.6)	2741 (9.2)	1.00		1.00	
No	2182 (75.4)	27,165 (90.8)	3.23 (2.95, 3.55)	<0.001	2.79 (2.48, 3.12)	<0.001
Number of children (32,896)						
>1	492 (17.1)	6350 (21.2)	1.00		1.00	
1	2301 (79.8)	23,163 (77.2)	0.78 (0.71, 0.86)	<0.001	1.02 (0.89, 1.17)	0.816
0	89 (3.1)	501 (1.7)	0.44 (0.34, 0.56)	<0.001	0.83 (0.50, 1.39)	0.481
Miscarriages (32,494)						
Yes	2018 (71.4)	20,794 (70.1)	1.00		1.00	
No	810 (28.6)	8872 (29.9)	1.06 (0.98, 1.16)	0.16	0.95 (0.86, 1.05)	0.350
Family history of cancer (33,057)						
Yes	540 (18.6)	5805 (19.3)	1.00		1.00	
No	2368 (81.4)	24,344 (80.7)	0.96 (0.87, 1.06)	0.370	0.91 (0.81, 1.03)	0.118

a Factors included in the multivariate model: region, age, ethnicities, education level, occupation, household income, marital status, age at marriage, health insurance, BMI, personal character, health condition, interpersonal relationship, major setback, number of children, miscarriages, family history of cancer, ever smoking, and alcohol consumption.

b Household income, low: <2000 RMB per month; medium: 2000–5000 RMB per month; high: >5000 RMB per month.

c Including unmarried, divorced, widowed, and separated.

### Association between overall life satisfaction and women's lifestyle

Overall life satisfaction is associated with healthy lifestyle. Compared to less satisfied women, satisfied women are less likely to be smokers (OR = 0.54; 95% CI: 0.47–0.62), more likely to do exercise (OR = 1.49; 95% CI: 1.26–1.75), and less likely to eat fried (OR = 0.60; 95% CI: 0.50–0.71), smoked (OR = 0.54; 95% CI: 0.47–0.63), pickled (OR = 0.66; 95% CI: 0.55–0.79), and grilled (OR = 0.63; 95% CI: 0.54–0.74) foods. Significant associations are not found between life satisfaction and alcohol consumption as well as diet pattern in multivariate analysis, though there are significant associations in the univariate analysis (Table 2).

**Table 2:** Association between life satisfaction and lifestyle in Chinese women

Variables (*N*)	Less satisfied (%) (2908)	Satisfied (%) (30,149)	Univariate		Multivariate^a^	
			OR (95% CI)	*P*	OR (95% CI)	*P*
Ever smoking (32,650)						
No	2497 (87.1)	26,616 (89.4)	1.00		1.00	
Yes	370 (12.9)	3167 (10.6)	0.80 (0.72, 0.90)	<0.001	0.54 (0.47, 0.62)	<0.001
Alcohol consumption (32,475)						
No	2719 (95.2)	28,509 (96.2)	1.00		1.00	
Yes	136 (4.8)	1111 (3.8)	0.78 (0.65, 0.94)	0.007	0.87 (0.71, 1.06)	0.155
Times of exercises/week (10,375)						
≤1	888 (78.9)	6487 (70.1)	1.00		1.00	
>1	237 (21.1)	2763 (29.9)	1.60 (1.37, 1.85)	<0.001	1.49 (1.26, 1.75)	<0.001
Diet pattern (10,374)						
More vegetable	734 (65.3)	5594 (60.5)	1.00		1.00	
Equal	357 (31.8)	3487 (37.7)	1.28 (1.12, 1.46)	<0.001	1.08 (0.93, 1.26)	0.334
More meat	33 (2.9)	169 (1.8)	0.67 (0.46, 0.98)	0.041	0.90 (0.58, 1.41)	0.648
Fried foods/week (9396)						
<1	205 (19.7)	2814 (33.7)	1.00	<0.001	1.00	<0.001
≥1	837 (80.3)	5540 (66.3)	0.48 (0.41, 0.56)		0.60 (0.50, 0.71)	
Smoked foods/week (9149)						
<1	367 (36.3)	4480 (55.1)	1.00	<0.001	1.00	<0.001
≥1	644 (63.7)	3658 (44.9)	0.47 (0.41, 0.53)		0.54 (0.47, 0.63)	
Pickled foods/week (9366)						
<1	186 (17.8)	2403 (28.9)	1.00	<0.001	1.00	<0.001
≥1	861 (82.2)	5916 (71.1)	0.53 (0.45, 0.63)		0.66 (0.55, 0.79)	
Grilled foods/week (9151)						
<1	319 (31.4)	3769 (46.3)	1.00	<0.001	1.00	<0.001
≥1	697 (68.6)	4366 (53.7)	0.53 (0.46, 0.61)		0.63 (0.54, 0.74)	

a Factors included in the multivariate model: region, age, occupation, household income, marital age, health insurance, character, health condition, interpersonal relationship, and major setbacks or unfortunate events.

### Overall life satisfaction and knowledge/examination of breast cancer

The percentage of women who have knowledge of breast cancer is 19.2% in the satisfied group and 14.7% in the less satisfied group (OR = 1.48; 95% CI: 1.29–1.70) (Table 3). However, numbers of sources of breast cancer knowledge show no significant difference between the satisfied group and the less satisfied group. Moreover, women satisfied with life are more likely to participate in regular physical examinations than those dissatisfied with life (OR = 1.11; 95% CI: 1.01–1.2).

**Table 3:** Association between life satisfaction and knowledge of breast cancer as well as physical examination in Chinese women

Variables (*N*)	Less satisfied (%) (2098)	Satisfied (%) (30,149)	Univariate		Multivariate^a^	
			OR (95% CI)	*P*	OR (95% CI)	*P*
Knowledge of breast cancer (32,984)						
No	712 (24.6)	5823 (19.4)	1.00		1.00	
A little	1758 (60.7)	18,504 (61.5)	1.29 (1.17, 1.41)	<0.001	1.36 (1.23, 1.51)	<0.001
Yes	424 (14.7)	5763 (19.2)	1.66 (1.47, 1.88)	<0.001	1.48 (1.29, 1.70)	<0.001
Sources of breast cancer knowledge (33,057)						
0	445 (15.3)	3956 (13.1)	1.00		1.00	
1	1596 (54.9)	15,714 (52.1)	1.11 (0.99, 1.24)	0.071	1.01 (0.89, 1.15)	0.896
2	612 (21.0)	7298 (24.2)	1.34 (1.18, 1.53)	<0.001	1.16 (1.00, 1.34)	0.050
≥3	255 (8.8)	3181 (10.6)	1.40 (1.20, 1.65)	<0.001	1.06 (0.89, 1.27)	0.524
Regular physical examination (32,689)						
No	1830 (63.5)	18,229 (61.2)	1.00		1.00	
Yes	1050 (36.5)	11,580 (38.8)	1.11 (1.02, 1.20)	0.012	1.11 (1.01, 1.22)	0.024

a Factors included in the multivariate model: region, age, occupation, household income, marital age, health insurance, character, health condition, interpersonal relationship, major setback or unfortunate events, and life satisfaction.

### Overall life satisfaction and breast cancer as well as benign breast diseases

Among the 33,057 participants, 102 were pathologically confirmed to have breast cancer, 90 cases in the satisfied group and 12 cases in the less satisfied group. Overall life satisfaction is significantly associated with lower detection rate of benign breast diseases (OR = 0.90; 95% CI: 0.82–0.99), and a lower but nonsignificant association with the detection rate of breast cancer (OR = 0.66; 95% CI: 0.35–1.25) (Table 4).

**Table 4:** Association between life satisfaction and detection rates of breast cancer and benign breast diseases in Chinese women

Variables (*N*)	Less satisfied (%) (2098)	Satisfied (%) (30,149)	Univariate		Multivariate^a^	
			OR (95% CI)	*P*	OR (95% CI)	*P*
Detection of benign breast disease (33,057)						
No	729 (25.1)	8857 (29.4)	1.00		1.00	
Yes	2179 (74.9)	21,292 (70.6)	0.80 (0.74, 0.88)	<0.001	0.90 (0.82, 0.99)	0.040
Detection of breast cancer (33,057)						
No	2896 (99.6)	30,059 (99.7)	1.00		1.00	
Yes	12 (0.4)	90 (0.3)	0.72 (0.40, 1.32)	0.291	0.66 (0.35, 1.25)	0.203

a Factors included in the multivariate model: area, age, occupation, household income, marital age, health insurance, character, health condition, interpersonal relationship, major setback or unfortunate events, ever smoking, alcohol consumption, and life satisfaction.

## Discussion

In this study, we found that women with higher overall life satisfaction were associated with significantly lower detection rate of benign breast diseases, and a lower, though nonsignificant detection rate of breast cancer. The inverse association may be explained, at least in part, by the positive association between overall life satisfaction and healthy lifestyle, knowledge of breast cancer, and regular physical examination.

It is well established that lifestyle play an important role on breast cancer risk. A large number of researches have evaluated the relationship between unhealthy lifestyle, including smoking, alcohol drinking, and unhealthy diet, and the risk of breast cancer. Recently, European Prospective Investigation into Cancer and Nutrition (EPIC) cohort reported a lower risk of breast cancer in women with healthy lifestyle combining healthy diet, never smoking, no alcohol consumption, and high physical activities 13. Alcohol drinking, especially in adolescence was supposed to increase the risk of proliferative BBD, 14, 15 and a healthy diet rich in fruits and vegetables could reduce proliferative diseases 16. However, the association between BBD and smoking 17, 18 as well as physical exercise 19, 20 remained controversial. In addition, the theory of Health Belief Model (HBM) 21 maintained that knowledge of disease had impacts on individual's health behaviors, such as compliance to preventative services and health care suggestions. Studies performing clinical breast examination (CBE) and mammogram screening showed that the more knowledge of breast cancer and more sources of health information women had, the more likely they would participate in screening 22, 23.

Previous studies were inconsistent on the association between life satisfaction and breast cancer risk. A null result was reported from the Finnish Twin Cohort comprising 12,032 women in Fenland, after 21 years of follow‐up 24. However, data from the Japan Collaborative Cohort (JACC) suggested that “ikigai”, a Japanese term meaning something that made one's life worth living, was associated with a significantly lower risk of breast cancer (RR = 0.66; 95% CI: 0.47–0.94) 25. A recent cross‐sectional study showed that life dissatisfaction might increase the risk of breast cancer for women in Eastern China 26, however, the information of life satisfaction was collected after the diagnosis of breast, which may bias the result of the study. It is worth noting that our study extend previous ones by indicating not only the possible inverse association between overall life satisfaction and breast diseases, but also suggest the potential explanations for the association. Moreover, to the best of our knowledge, we are the first to confirm the inverse association between overall life satisfaction and benign breast diseases. The nonsignificant association between overall life satisfaction and breast cancer is largely due to the small number of cases identified in this study.

In our study, 19.8% were reported to have no knowledge about breast cancer, and this proportion is comparable to the 19.4% reported in a cross‐sectional study conducted in Eastern China, which also identified association between overall life satisfaction and knowledge of breast cancer 27. We assumed that life satisfaction indirectly protected women from breast diseases by affecting knowledge of breast cancer or physical examinations. In addition, it was plausible that knowledge of breast cancer might have impacts on women's health behaviors (smoking, alcohol consumption, healthy diet, and physical exercises).

Generally, our study found a healthier lifestyle (less smoking and unhealthy food, more exercises) among women satisfied with their overall lives. Previous studies also showed certain psychosocial factors might affect the behavior of smoking 28, alcohol consumption 29, diet 30, and physical activities 31, 32, 33. The association between life satisfaction and lifestyle can be explained from two aspects: on one hand, dissatisfied women may have more stress or negative attitude to deal with problems and events in life, which may lead to pessimistic emotions and subsequently result in unhealthy lifestyle, like smoking and alcohol drinking 28, 34; on the other hand, satisfied women have more confidence or self‐efficacy with life, and the positive effects may motivate them to do more physical exercises 32.

We acknowledge that this is a cross‐sectional study and there are intrinsic limitations in explanation for temporal relationships between a low overall life satisfaction and late age at marriage, interpersonal relationship, and most unhealthy lifestyle in Table 3. Life satisfaction was first used in social psychology and often measured by scales, such as Life Satisfaction Rating Scale (LSR) and Satisfaction With Life Scale (SWLS). However, because of a large number of questions, most scales were not suitable for epidemiological questionnaires. In our study, overall life satisfaction was measured using a single item with five levels and this measure was widely used in other literatures 35, 36 and it was reported that life satisfaction was relatively stable in adult 37. In addition, we randomly selected 5% of the questionnaires for replication, and the quality was found to be high. Furthermore, from Table 1, the relationship between variables and life satisfaction are plausible, this is also a reflection of the good quality for life satisfactory measurement.

Overall life satisfaction is a comprehensive index related to almost any aspect of life. In our study, overall life satisfaction is related to factors including region, age, age at marriage, occupation, household income, insurance, character, international relationship, health condition, and major unfortunate events. Therefore, an overall well‐being of women will be beneficial to the protection of breast diseases. Further prospective studies are warranted to investigate the association between overall life satisfaction and breast cancer.

## Conflict of Interest

The authors declare that they have no competing interests.
